# Theoretical Study on the Difference in Electron Conductivity of a One-Dimensional Penta-Nickel(II) Complex between Anti-Ferromagnetic and Ferromagnetic States—Possibility of Molecular Switch with Open-Shell Molecules

**DOI:** 10.3390/molecules24101956

**Published:** 2019-05-21

**Authors:** Yasutaka Kitagawa, Hayato Tada, Iori Era, Takuya Fujii, Kazuki Ikenaga, Masayoshi Nakano

**Affiliations:** 1 Department of Materials Engineering Science, Graduate School of Engineering Science, Osaka University, Toyonaka, Osaka 560-8531, Japan, hayato.tada@cheng.es.osaka-u.ac.jp (H.T.); iori.era@cheng.es.osaka-u.ac.jp (I.E.); tfujii@cheng.es.osaka-u.ac.jp (T.F.); kazuki.ikenaga@cheng.es.osaka-u.ac.jp (K.I.); 2 Center for Spintronics Research Network (CSRN), Graduate School of Engineering Science, Osaka University, Toyonaka, Osaka 560-8531, Japan; 3 Quantum Information and Quantum Biology Division, Institute for Open and Transdisciplinary Research Initiatives, Osaka University, Toyonaka, Osaka 560-8531, Japan; 4 Institute for Molecular Science, 38 Nishigo-Naka, Myodaiji, Okazaki 444-8585, Japan

**Keywords:** EMACs, one-dimensional penta-nickel (II) complex, electron conductivity, density functional theory, elastic scattering Green’s function theory

## Abstract

The electron conductivity of an extended metal atom chain (EMAC) that consisted of penta-nickel(II) ions bridged by oligo-α-pyridylamino ligands was examined by density functional theory (DFT) and elastic scattering Green’s functions (ESGF) calculations. The calculated results revealed that an intramolecular ferromagnetic (FM) coupling state showed a higher conductivity in comparison with an anti-ferromagnetic (AFM) coupling state. The present results suggest the potential of the complex as a molecular switch as well as a molecular wire.

## 1. Introduction

A technology to manipulate single molecules has been developing to realize molecule-based devices. At the same time, it is necessary to find more suitable molecules for such devices [1,2,3]. From the viewpoint of molecule-based devices such as wires, switches, and transistors, it is crucial to control electronic conductivities by external stimuli, i.e., light irradiation, electronic fields, and so on [4]. As an example, it has been reported that open-shell molecules, such as single-molecule magnets, can be utilized to realize a molecular switch by external magnetic fields [5,6].

Linearly aligned polynuclear metal complexes that are called extended metal atom chains (EMACs) are one of the most promising materials for “molecular wires”. Many reports about their properties and synthesis can be found in the literature [7,8,9,10,11,12,13,14,15]. Up to now, Peng and co-workers have succeeded in the synthesis of many noble EMACs by using oligo-α-pyridylamino ligands, such as [M_5_(tpda)_4_X_2_], [M_7_(teptra)_4_X_2_], [Ni_11_(tentra)_4_Cl_2_](PF_6_)_4_ (tpda: tripyridyldiamine, teptra: tetrapyridyldiamine, tentra: tetra-naphthyridyltri-amide, M: Cr, Co, Ni, etc., and X : Cl, CN, N_3_, NCS, etc.) [16,17,18,19]. They have also reported the magnetic properties of those complexes in addition to their ability of electron conductivities [20,21]. Very recently, for example, they succeeded in measuring the magnetic properties and electron conductance of the undeca-nickel metal string complex; [Ni_11_(bnatpya)_4_Cl_2_]^4+^ [21]. In addition, Huang and co-workers have also reported a negative differential resistance (NDR) behavior of the hetero-nuclear Ni_3_Ru_2_ complex [22]. These EMACs, therefore, are promising open-shell systems that have a potential for single-molecule devices. 

**Figure 1:**
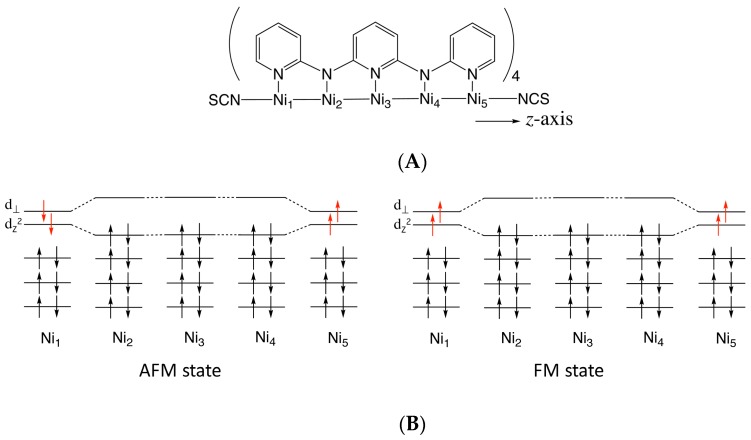
(**A**) Illustration of the molecular structure of [Ni_5_(tpda)_4_(NCS)_2_] (tpda: tripyridyldiamine) (**1**). The *z*-axis was fixed along the chain axis. (**B**) Spin structure of the anti-ferromagnetic (AFM) and ferromagnetic (FM) states of complex **1**. The d_z_^2^ orbital becomes one of the magnetic orbitals. Because of the helical structures of the oligo-α-pyridylamino ligands, another magnetic orbital (d_⊥_) is expressed by hybridization of *d_x_^2^-_y_^2^* and *d_xy_* orbitals.

On the other hand, as a development of computational procedures to calculate properties of the single-molecule, the electron conductivities of various magnetic molecules have been estimated by theoretical calculations [23,24]. Our group also has investigated the magnetic properties and electron conductivities of a series of the EMACs that consist of nickel(II) ions bridged by oligo-α-pyridylamino ligands by density functional theory (DFT) and elastic scattering Green’s functions (ESGF) methods [25,26,27]. The experimental studies have already revealed that there are two types of nickel(II) ions in the complexes, i.e., terminal (*S* = 1) and inner (*S* = 0) ions, where *S* represents the spins of each nickel(II) ion. It has also been found that those terminal nickel ions are weakly coupled in an anti-ferromagnetic (AFM) manner in the chain (see Figure 1B) [20]. We have reported that the DFT and ESGF calculations can reproduce the experimental results of the intra-molecular magnetic interactions and electron conductivities [26,27]. Very recently, in addition, we found that the electron conductivity of the tri-nickel(II) complex [Ni_3_(dpa)_4_(NCS)_2_] strongly depends on its intra-molecular spin-coupling states [28]. The spin polarization due to the strong electron correlation effect and the induced orbital localization decreases the electron conductivity in the AFM state. As a result, the ferromagnetic (FM) coupling state shows a conductivity 89 times higher than that of the AFM state. The result indicates the potential of the open-shell EMACs as molecular switches controlled by the external magnetic field. The energy gap between the AFM and FM states (ca. 673 cm^−1^), however, is too large to allow control of the spin states by the external field. One possibility to overcome the problem is to elongate the chain length to separate two terminal nickel(II) ions in order to weaken the anti-ferromagnetic coupling. In this study, therefore, we focused on the penta-nickel(II) complex [Ni_5_(tpda)_4_(NCS)_2_] (**1**). First, we examined the electronic structures and electron conductivities of the AFM and FM states. 

The conductivity of the FM state was obviously different from that of the AFM state reported in previous paper [27]. Furthermore, the potential as a molecular switch of the penta-nickel(II) complex appeared to be promising in comparison with that of the tri-nickel(II) complex. Finally, we discuss some designing guidelines to realize the most effective molecular switch by using the spin state of open-shell molecules.

## 2. Theoretical Background and Computational Details

### 2.1. Calculation of Electron Conductivity of Open-Shell Systems

The conductivity of small systems is usually evaluated by a non-equilibrium Green’s function (NEGF) method using molecular orbitals obtained from, for example, extended-Hückel, DFT, and other quantum chemical calculations [23,24]. Because the penta-nickel(II) complex is an open-shell system with localized spins, we considered the open-shell (spin-polarized) electronic structures [26,27]. In order to make a simple formulation and to connect to the broken-symmetry calculations for open-shell systems, we focused on the ESGF method that was originally proposed by Mujica and Luo and improved it for a spin-polarized system [25,29,30,31,32]. In this section, the theoretical background of the extended ESGF method is briefly explained. 

**Figure 2:**
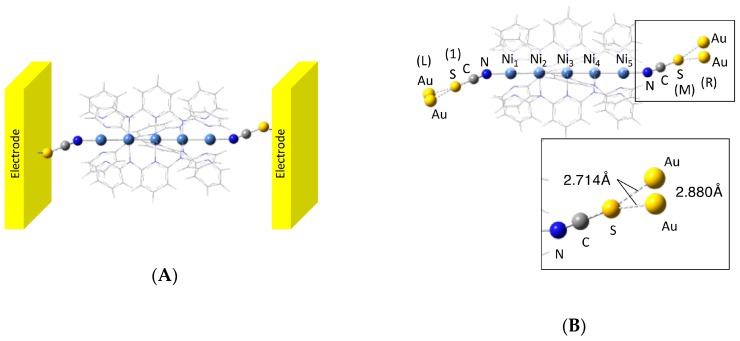
(**A**) Illustration of complex **1** connected to gold electrodes. (**B**) Calculated model system **1** consisting of complex **1** and Au_2_. The labels 1, M, L, and R in parentheses correspond to those in Equations (1)–(8).

Let us consider that complex **1** is connected to gold electrodes by the sulfur atoms of NCS ligands, as illustrated in Figure 2A. It has been confirmed that the gold electrodes can be simply approximated to gold dimers [27]; therefore, we considered a system in which complex **1** was connected to the gold dimers i.e., an extended molecular system, as illustrated in Figure 2B. The extended molecular system of the AFM and FM states are abbreviated **1_AFM_** and **1_FM_**, respectively. When a voltage (*V*_D_) is applied to the right (R) and left (L) electrodes using the extended ESGF method, the static carrier conduction of the system ($i_{\sigma }^{LR}$) between L and R for a spin (σ) is given by

(1) $$i_{\sigma }^{LR}=\frac{1}{2}\underset{n}{\sum }\frac{emk_{B}T}{2\pi^{2}h^{3}}\int_{eV_{D}}^{\infty }dE{\left|T_{\sigma }\left(E\right)\right|}^{2}f_{\sigma }\left(E\right),$$

and

(2) $$f_{\sigma }\left(E\right)=\left\{\ln\left[1+\exp\left(\frac{E_{F,\sigma }+eV_{D}-E}{k_{B}T}\right)\right]-\ln\left[1+\exp\left(\frac{E_{F,\sigma }-E}{k_{B}T}\right)\right]\right\},$$

where $E_{F}$ and *T* are the Fermi energy and temperature, respectively. Constants$\;k_{B}$ *e*, *m*, and *h* are Boltzmann constant, elementary charge, invariant mass of electron, and Planck constant, respectively. Here, we assumed that the middle energy values of the highest occupied molecular orbital (HOMO) and the lowest unoccupied molecular orbital (LUMO) of the extended molecular system corresponded to the Fermi energy. In the extended ESGF method, a square of the transition probability for the spin σ at a given voltage *E*; ${\left|T_{\sigma }\left(E\right)\right|}^{2}$ can be expressed by

(3) $${\left|T_{\sigma }\left(E\right)\right|}^{2}={\left(\gamma_{L1,\sigma }\gamma_{MR,\sigma }\right)}^{2}\underset{p}{\sum }\frac{{|\langle 1|\phi_{p,\sigma }|\rangle }^{2}{\left|\langle \phi_{p,\sigma }|M\rangle \right|}^{2}}{{\left(\varepsilon_{p,\sigma }-E\right)}^{2}+\Gamma_{p,\sigma }{}^{2}},$$

where $\Gamma_{p,\sigma }$ denotes a spin-dependent escape rate determined by the Fermi’s golden rule, i.e.,

(4) $$\Gamma_{p,\sigma }=\frac{\gamma_{L1,\sigma }\langle 1|\phi_{p,\sigma }\rangle +\gamma_{MR,\sigma }\langle \phi_{p,\sigma }|M\rangle }{2}.$$

Here, $\left\{|\phi_{p,\sigma }\right\}$ was approximated by Kohn–Sham orbitals obtained from the spin unrestricted (or broken-symmetry) DFT calculations of the whole extended molecular system. In this paper, we used 20 orbitals (HOMO-9 – LUMO+9) for the calculation of the conductivity. The parameter $\gamma_{X}$ (X = L1, σ and MR, σ) is proportional to the transition probability from the electrode to the complex. To determine $\gamma_{X}\;$, the occupied orbitals of the electrodes are assumed to interact with the LUMO of the complex. Therefore, $\gamma_{X}\;$can be represented by

(5) $$\gamma_{L1,\sigma }=V_{L,\sigma }\left(\mathrm{LUMO}\right)d_{1,\sigma }\left(\mathrm{LUMO}\right),$$

(6) $$V_{L,\sigma }^{2}\left(\mathrm{LUMO}\right)=\frac{\left(\Delta E_{\sigma ,\mathrm{HOMO}-\mathrm{LUMO}}-\Delta E_{\sigma ,\mathrm{LUMO}}\right)\Delta E_{\sigma ,\mathrm{LUMO}}}{2},$$

(7) $$d_{1,\sigma }^{2}\left(\mathrm{LUMO}\right)=\frac{\sum_{i}c_{1,i,\sigma }^{2}}{\sum_{a,j}c_{a,j,\sigma }^{2}},$$

where $\Delta E_{\sigma ,\mathrm{HOMO}-\mathrm{LUMO}}$ is the HOMO–LUMO gap of the whole system, and $\Delta E_{\sigma ,\mathrm{LUMO}}$ is the energy difference between the HOMO of the isolated Au dimers and the LUMO of the isolated complex; $d_{1,\sigma }\left(\mathrm{LUMO}\right)$ is a weight parameter of the atomic orbital (AO) coefficient of sulfur 1 of the axial NCS ligand in the LUMO, where $c_{n,m,\sigma }$ is an orbital coefficient of the *m-*th basis set of atom *n* for spin σ. In the case of complex **1**, however, the LUMO and LUMO+1 were quasi-degenerate as explained below, so that we also included the effect of those quasi-degenerate orbitals by weighting with the Boltzmann factor. The effective strength of $d_{1,\sigma }$ is approximately averaged as

(8) $$d_{1,\sigma }=\frac{\sum_{k}B_{k}d_{LUMO+k}}{\sum_{k}B_{k}},$$

where *k* represents the number of the degenerate orbitals around LUMO, and *B*_k_ is the Boltzmann factor, with the temperature assumed to be 300 K. In order to depict the I–V characteristics, voltage from 0.1 to 3 V was applied. Here, we noted that there might be a possibility that the applied maximum voltage (3V) was over the breakdown voltage. 

### 2.2. Computational Details

For the calculations of the electronic structures of the penta-nickel(II) complex, we constructed a model structure using the atomic coordinates of X-ray crystal structural analysis [16]. The electrodes approximated by the Au dimers were attached to the NCS ligands as shown in Figure 2B. It was confirmed that the Au dimer reproduces the Fermi energy of the bulk gold [27]. The Cartesian coordinates of the model system **1** for the extended molecular system are summarized in Table S1 in Supplementary Materials. Using the model structure **1**, we performed DFT calculations for both AFM and FM states (abbreviated as **1_AFM_** and **1_FM_**, respectively). For the spin-polarized electronic structures, all calculations were performed using the spin-unrestricted DFT method with the Becke 3-parameter Lee–Yang–Parr hybrid functional set (UB3LYP). As a basis sets, Tatewaki–Huzinaga MIDI plus p-type (533(21)/53(21)/(41)), 6-31+G*, and 6-31G were used for Ni atoms, axial ligand atoms, and bridging ligand atoms, respectively. All DFT calculations were performed in the gas phase by using Gaussian09 [33]. For calculations of the conductivity, we used our self-developed program.

## 3. Calculated Results 

### 3.1. Electronic Structures 

**Figure 3:**
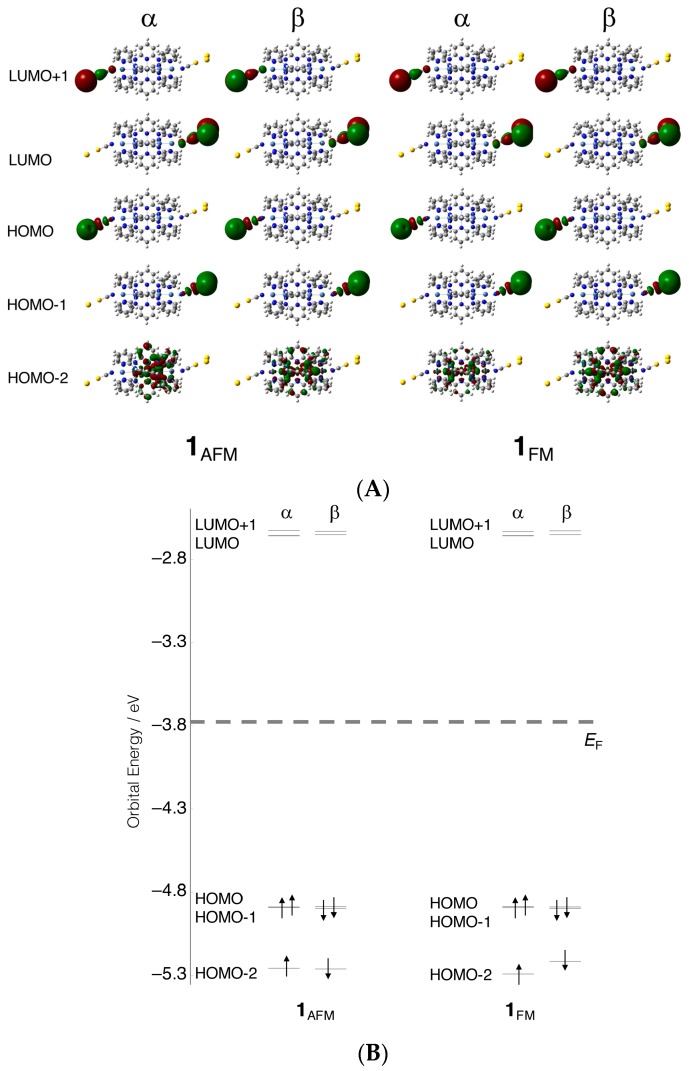
(**A**) Calculated frontier orbitals (highest occupied molecular orbital (HOMO)-2, to lowest unoccupied molecular orbital (LUMO)+1) and (**B**) their energies of **1_AFM_** and **1_FM_**.

At first, the open-shell electronic structures of **1_AFM_** and **1_FM_** were confirmed. The calculated frontier orbitals (HOMO-2 – LUMO+1) and their energies are illustrated in Figure 3. The HOMO–LUMO energy gaps (Δ_H-L_) and estimated Fermi energies (*E*_F_) for these models are summarized in Table 1. Other orbital information for HOMO-9–LUMO+9 that were used for the calculation of electron conductivities is summarized in Figure S1 and Table S2. Pairs of “HOMO and HOMO-1” and “LUMO and LUMO+1” were shown to be quasi-degenerate, and their energies were found to be around −4.89 and −2.65 eV, respectively, in both models. Fermi energies defined by the middle value between HOMO and LUMO energies were, therefore, estimated about −3.77 eV. This value was higher than *E*_F_ value of Au_2_ (ca. −5.6 eV) [27]. 

This destabilization of *E*_F_ was considered to originate from an anti-bonding interaction between Au_2_ and the sulfur atom of the NCS ligand. In both **1_AFM_** and **1_FM_**, as shown in Figure 3A, the quasi-degenerate HOMOs were σ-type anti-bonding orbitals, while the quasi-degenerate LUMOs were π-type orbitals between Au_2_ and the NCS ligand. The Δ_H-L_ values around 2.23–2.24 eV were typical of a semi-conductor [34]. The characteristics of the frontier orbitals of **1_FM_** and **1_AFM_** were quite similar, and furthermore, the Δ_H-L_ values of the penta-nickel(II) complex were almost equivalent to the values of the tri-nickel(II) complex [28]. This suggests that the frontier orbitals and the energies of both tri- and penta-nickel(II) complexes were governed by characteristics of the axial NCS ligands in the FM and AFM states, as shown in Figure 3A. Meanwhile, the orbitals consisting of nickel atomic orbitals were found below/above the quasi-degenerate HOMOs and LUMOs. For example, occupied nickel dσ orbitals could be found in HOMO-2 about 0.3–0.4 eV lower than in HOMO-1. In this way, the spin-polarized dσ and dδ orbitals that were responsible for the magnetic properties, were divided into upper and lower levels. Consequently, the singly occupied molecular orbitals (SOMOs) of the FM state and the spin-polarized magnetic d-orbitals of the AFM state were energetically separated from the frontier orbitals. This phenomenon was also found in the tri-nickel(II) complex, but the energy splitting between HOMO-1 (NCS–Au_2_ anti-bonding orbital) and HOMO-2 (dσ orbital) of the penta-nickel(II) complex was smaller than the corresponding values of the tri-nickel complex (ca. 0.7 eV), along with the elongation of the chain length [28]. 

In order to show the spin structures of **1_AFM_** and **1_FM_**, the atomic spin densities are also summarized in Table 1. The spin densities were only found in the terminal nickel ions, even though the gold dimers were attached to the complex, and the absolute values were almost equivalent to those of the isolated complex **1** [27]. As depicted in Figure 1, two spins were formally localized at the terminal nickel(II) ions [20]. Therefore, the atomic spin densities of the terminal nickel(II) should be 2.0 (or –2.0 for β spin). The calculated values, however, were slightly smaller than the formal values. This phenomenon originated from the delocalization of the magnetic orbitals. There were two magnetic orbitals, i.e., *d_z_*^2^ and *d*_⊥_, as illustrated in Figure 1, because *d*_⊥_ consisted of *d_x_*^2^*_-y_*^2^ and d*_xy_* orbitals that showed no delocalization along with *z*-axis. On the other hand, *d_z_*^2^ orbitals had lobes along the *z*-axis; therefore, they easily delocalized along the one-dimensional axis. This delocalization provided an overlap between α and β orbitals, as reported in reference [26]. In reference [26], it was reported that if there is a small overlap between α and β *d_z_*^2^ orbitals, then the spin densities on each terminal nickel(II) slightly decrease. A small portion of spin densities on Ni_2_ and Ni_4_ was also considered to originate from this delocalization of *d_z_*^2^ orbitals. In addition, excess spin densities found in ligands (0.445) were widely delocalized on the whole ligands; nevertheless, the absolute values on each atom were quite small. The detailed spin densities of the whole system are summarized in Table S4.

As already reported [26,27], gold dimers hardly affected the spin structures of the complex, and the spins remained localized at Ni_1_ and Ni_5_, as illustrated in Figure 1B. In other words, the AFM and FM states were open-shell systems that can be described by the Heisenberg Hamiltonian two-spin-site model;

(9) $$H=-2J{\hat{S}}_{{\mathrm{Ni}}_{1}}\cdot {\hat{S}}_{{\mathrm{Ni}}_{5}},$$

where ${\hat{S}}_{{\mathrm{Ni}}_{1}}$ and ${\hat{S}}_{{\mathrm{Ni}}_{5}}$ are spin operators for the spin sites on both ends, i.e., Ni_1_ and Ni_5_, respectively, and *J* is an effective exchange integral between them. The *J* value was estimated by the energy gap between **1_AFM_** and **1_FM_** by using Yamaguchi equation [35,36],

(10) $$J=\frac{E^{AFM}-E^{FM}}{\langle {\hat{S}}^{2}\rangle {}^{FM}-\langle {\hat{S}}^{2}\rangle {}^{AFM}}.$$

The calculated *J* value was −10.2 cm^−1^, showing a weak anti-ferromagnetic coupling between Ni_1_ and Ni_5_. The value was consistent with that of the isolated complex (–9.5 cm^−1^) [27], indicating the negligible contribution of the Au cluster to the magnetic interaction. On the other hand, the value was much smaller than the value of the tri-nickel(II) system (−110.8 and −112.3cm^−1^ for the isolated complex and extended molecular system, respectively) [27,28], so that the estimated energy gap between **1_AFM_** and **1_FM_** (41 cm^−1^) was much smaller than the values of the tri-nickel(II) complex (ca. 450 cm^−1^) [27] .

### 3.2. Electron Conductivity

**Figure 4:**
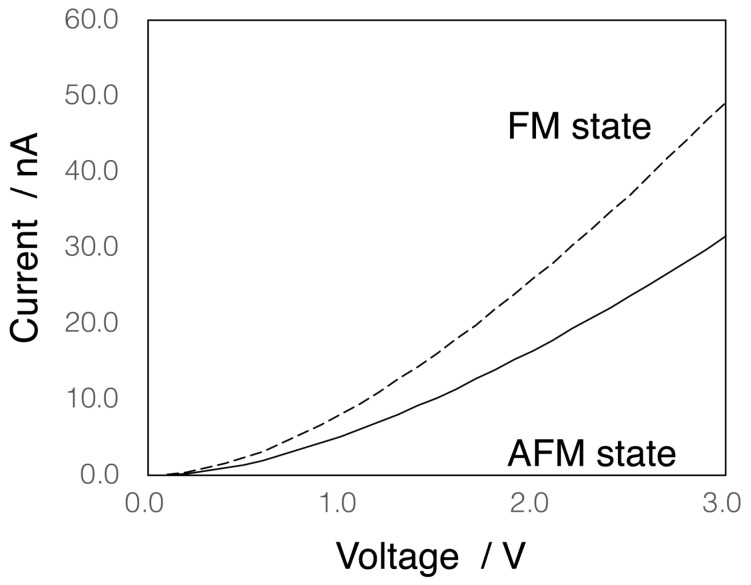
Calculated I–V characteristics. Solid and dashed lines represent **1_AFM_** and **1_FM_**, respectively. The applied voltage was assumed to be in the range from 0 to 3 V.

**Figure 5:**
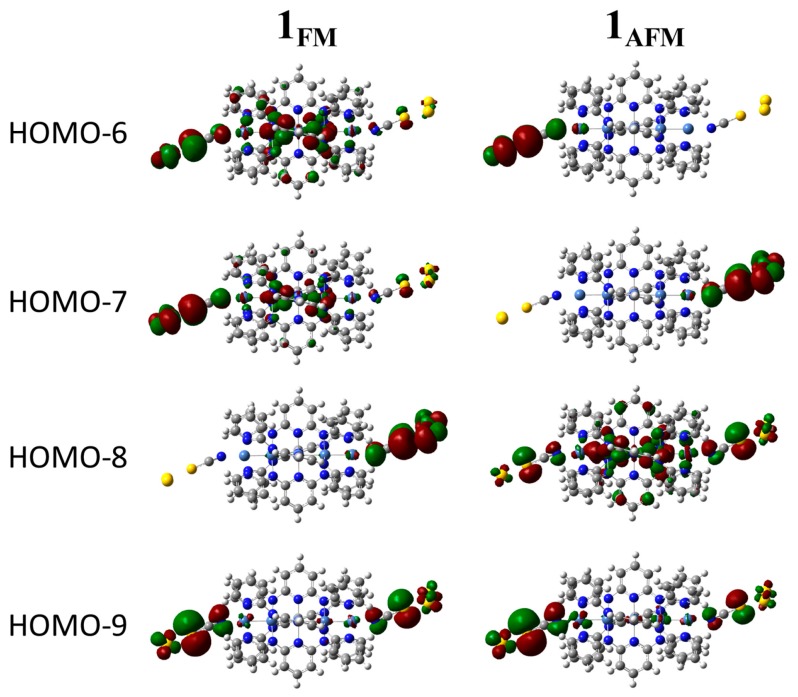
From α-HOMO-6 to α-HOMO-9 of **1_FM_** and **1_AFM_**.

We next evaluated the electron conductivities of both **1_AFM_** and **1_FM_** by the ESGF method using the above orbitals and their energies. The obtained I–V characteristics are shown in Figure 4. The plotted curve of the ground state, **1_AFM_**, well reproduces the characteristics of single-molecule electric conduction, and is also in good agreement with experimental results [23], although the extended ESGF approach would not be enough for quantitative analyses in comparison with other approaches using, for example, Landauer–Bütikker formulation [23]. The calculated current value of **1_AFM_** at 1V (5.1 nA) was consistent with the experimental values (ca. 7 nA [21]), indicating that the ESGF approach is reliable semi-quantitatively, as already reported [27]. As explained above, the electron conductivity is roughly described by three parameters, i.e., “overlaps between electrode and S atom ($\gamma_{X}$)”, “site-overlap (${\left|\langle 1|\phi_{p,\sigma }\rangle \right|}^{2}{\left|\langle \phi_{p,\sigma }|M\rangle \right|}^{2}$ in Equation (3))”, and “energy differences between orbital energies and *E*_F_”. In a previous paper [28], we reported that the site-overlap values of dσ orbitals dominantly contribute to the electron conductivity in the case of the tri-nickel(II) complex. As summarized in Table S3, HOMO-8 and HOMO-9 that are σ-type Ni(II) orbitals, as depicted in Figure 5, indeed showed significant site overlaps. Those were, therefore, considered to dominantly contribute to the conductivity of **1_AFM_**. 

On the other hand, the current value of **1_FM_** was 8.0 nA at 1V, that is, 1.6 times larger than the value of **1_AFM_**. As summarized in Table S3, however, we could not find significant differences in the $\gamma_{X}$ values that were found to lie in a range from 0.145 to 0.174 between the AFM and FM states. This is easily understood from Figure 3B, in which LUMOs are almost equivalent in those two states. In contrast, the site-overlap values changed according to the spin states. For example, the HOMO-6 and HOMO-7 of **1_FM_** exhibited significantly larger site-overlap values, while the corresponding orbitals of **1_AFM_** were almost zero. As depicted in Figure 5, those orbitals of **1_FM_** that dominantly consisted of σ-type orbitals of Ni(II) ions hybridizing with π-type anti-bonding orbitals between NCS ligands and Au atoms were widely delocalized to S atoms of both ends. In the case of **1_AFM_**, the corresponding orbitals tended to be localized at each end. Consequently, **1_FM_** showed higher conductivity in comparison with **1_AFM_**.

As shown above, a difference in electron conductivity between the AFM and FM states dominantly originated from site overlap; in other words, molecular orbitals of the complex **1** delocalized to S atoms of axial NCS ligands. Those results indicated that a degree of orbital hybridization between metal d-orbitals and axial ligand orbitals is important for the higher conductivity of EMACs. Here, let us consider two fragments of MOs, $\psi_{metal}$ and $\psi_{ligand}$, for metal and axial ligand orbitals, respectively. For orbital hybridization between those orbitals ($c_{metal}\psi_{metal}+c_{ligand}\psi_{ligand}$), the orbital overlaps and orbital energy difference between them are important. $\psi_{metal}$ and $\psi_{ligand}$ should lie on similar energy levels and have a larger overlap to realize sufficient orbital hybridization coefficients *c*. In addition, the axial ligand orbitals in HOMO-6 and HOMO-7 of **1_FM_** were π-type orbitals between NCS ligand and Au atoms; nevertheless, the corresponding metal orbitals were dσ orbitals. Usually, Au ligand antibonding σ-type orbitals are higher in energy than dσ-orbitals [27]. Therefore, those pσ-type orbitals of the ligands hardly hybridized with dσ-orbitals and were found to be localized degenerate HOMOs (see Figure 3B). Conversely, it was expected that much higher conductivities be realized if the pσ-orbital energies of the axial ligands were tuned to be comparable to the dσ-orbitals or by using other axial ligands with appropriate orbital energies. 

## 4. Conclusions

In this paper, we examined electron conductivities of EMAC consisting of penta-nickel(II) ions bridged by oligo-α-pyridylamino ligands by the DFT and ESGF calculations. We especially analyzed the calculated results in terms of the difference in electron conductivities between the spin states. The calculated results indicated that the conductivity of **1_FM_** was higher than that of **1_AFM_**. However, the absolute value was smaller than the value of the FM state of the tri-nickel(II) complex [28]. The difference in the conductivities was considered to originate from the difference in their chain lengths. The distances between terminal nickel ions of [Ni_3_(dpa)_4_(NCS)_2_] and [Ni_5_(tpda)_4_(NCS)_2_] were 4.86 and 9.33 Å, respectively [20], and the elongation of the chain length led to a decrease in the site overlap. On the other hand, the elongation between the spin sites obviously decreased the energy gap between the AFM and FM states. As a consequence, the conductivity of the penta-nickel(II) complex was considered to be easily increased by the increase of the population in the FM state with thermal or magnetic stimuli. In this sense, the penta-nickel complex has potential as a molecular switch and/or a molecular transistor in which the conductance can be controlled by the external field. In this paper, we simulated the conductivity at 300K. This temperature is considered to be high enough in comparison with the estimated energy gap between **1_AFM_** and **1_FM_**. The two spin states, therefore, coexisted because of a Boltzmann distribution at 300K, so that the real conductance of the extended molecular system was considered to be the averaged value of the two states. So, one must perform the experiment under the much lower temperature condition. By decreasing the temperature, the conductance is decreased because the population of the ground state, i.e., the AFM state that indicates lower conductance, becomes dominant. Then, a change in the conductance by changing the spin states into the FM state appears more significantly. 

It is, however, necessary to increase the total electron conductivity and differentiate the electron conductance between the AFM and FM states for a more reliable molecular switch, suggesting a necessity of a rational molecular design based on quantum chemistry. For example, our results indicate the importance of selection of the axial ligand species and of the metal ions for higher conductivity. By using the axial ligands with the same orbital energy level as that of the dσ orbitals, the conductivity of the system was considered to be raised because the site-overlap increased, as mentioned above. In addition, it is, of course, crucial to decrease the HOMO–LUMO energy gap. On the other hand, it was also reported that the orbital overlap between metal ions can be controlled by the functional groups of the bridging ligands [37], suggesting the possibility that the site overlaps, and therefore, the conductivities of AFM and FM states can also be tuned.

In this way, our studies about the relationship between electronic structures, spin structures, and electron conductivities of nickel EMACs have obviously indicated a difference in the conductivities between spin states. In conclusion, the open-shell EMACs are considered to be promising materials for molecular switches as well as for molecular wires, and a rational investigation based on quantum chemical calculations is a powerful approach for their molecular design.

## Figures and Tables

**Table 1:** Summary of calculated values for **1_AFM_** and **1_FM._** ^a^

	1_AFM_	1_FM_
*E*_F_/eV	α −3.77/β −3.77	α −3.77/β −3.77
Δ_H-L_/eV	α 2.23/β 2.24	α 2.23/β 2.24
Atomic spin densities/atomic units ^b^		
Ni_1_	−1.592	1.593
Ni_2_	−0.114	0.120
Ni_3_	0.000	0.034
Ni_4_	0.109	0.116
Ni_5_	1.595	1.596
Sum of NCS ligands	0.001	0.104
Sum of gold dimers	0.000	−0.008
Other ligands	0.001	0.445
Total energy/atomic unit	−12470.68913	−12470.68895
${\hat{S}}^{2}$	2.0201	6.024

^a^ α and β represent values of α and β orbitals, ^b^ see Figure 2B.

## Supplementary Materials

The following are available online at https://www.mdpi.com/1420-3049/24/10/1956/s1, Table S1: Cartesian coordinate of the model structure, Table S2: Calculated orbital energies of **1_AFM_** and **1_FM_** systems, Table S3: Calculated $\gamma_{L1}\gamma_{MR}$ and site-overlap values of **1_AFM_** and **1_FM_** systems, Figure S1: Illustrations of LUMO+9 to HOMO-9 of **1_AFM_** and **1_FM_**.
