# Supplementary material for: Theoretical Study on the Difference in Electron Conductivity of a One-Dimensional Penta-Nickel(II) Complex between Anti-Ferromagnetic and Ferromagnetic States—Possibility of Molecular Switch with Open-Shell Molecules
Source: Molecules. 2019 May 21;24(10):1956. doi: 10.3390/molecules24101956 (PMC6571866; doi:10.3390/molecules24101956)
Supplement: Supplementary file 1 [file molecules-24-01956-s001.pdf]

Supplementary Materials for

Theoretical study on difference in electron conductivity of one-dimensional penta-nickel(II) complex between anti-ferromagnetic and ferromagnetic states – Possibility of molecular switch with open-shell molecules

Yasutaka Kitagawa [1,2,*], Hayato Tada [1], Iori Era [1], Takuya Fujii [1], Kazuki Ikenaga [1], Masayoshi Nakano [1,2,3,4,*]

[1] Department of Materials Engineering Science, Graduate School of Engineering Science, Osaka University, Toyonaka, Osaka 560-8531, Japan.
[2] Center for Spintronics Research Network (CSRN), Graduate School of Engineering Science, Osaka University, Toyonaka, Osaka 560-8531, Japan.
[3] Quantum Information and Quantum Biology Division, Institute for Open and Transdisciplinary Research Initiatives, Osaka University, Toyonaka, Osaka 560-8531, Japan.
[4] Institute for Molecular Science, 38 Nishigo-Naka, Myodaiji, Okazaki 444-8585, Japan.
* Correspondence: (Yasutaka Kitagawa) e-mail : kitagawa@cheng.es.osaka-u.ac.jp; (Masayoshi Nakano) e-mail : mnaka@cheng.es.osaka-u.ac.jp

Table S1. Cartesian coordinate of the model structure (in Å)

Atom	*x*	*y*	*z*	Atom	*x*	*y*	*z*
Ni	0.00102	0.05401	-2.36645	H	-4.11473	2.72929	-3.55977
Ni	0.00000	0.00000	0.00000	H	-5.18201	2.56876	-1.51296
Ni	0.00000	0.00000	2.29849	H	-4.28957	1.24509	0.17323
Ni	0.00411	0.00000	4.59278	N	1.90919	-0.77182	-2.12065
Ni	0.07465	0.00000	6.96305	C	2.55583	-0.69769	-0.91854
N	-0.02910	0.11090	-4.36366	N	1.88496	0.00693	0.07015
C	-0.11999	0.52434	-5.42944	C	2.55118	0.58576	1.15451
S	-0.32514	1.13707	-6.92938	N	1.78539	0.66029	2.28656
N	0.17337	-0.04456	8.95920	C	2.30944	1.19444	3.46020
C	0.40542	-0.43732	10.01812	N	1.41410	1.24670	4.51332
S	0.79641	-0.98607	11.48874	C	1.54596	2.19645	5.52988
N	-1.81337	0.92803	6.74758	N	0.94874	1.85036	6.70327
C	-2.18727	1.45286	5.54984	C	0.95412	2.75219	7.70397
N	-1.27246	1.39566	4.55148	C	1.52044	3.97538	7.62756
C	-1.29603	2.24599	3.46129	C	2.13225	4.30350	6.45376
N	-0.71275	1.76863	2.32256	C	2.12720	3.44615	5.37771
C	-0.67227	2.50935	1.17330	H	2.51019	3.69358	4.54428
N	-0.04106	1.88564	0.10256	H	2.56878	5.14466	6.38745
C	0.62837	2.60464	-0.85949	H	1.50632	4.59698	8.34583
N	0.78568	1.96618	-2.07203	H	0.53961	2.54420	8.53307
C	1.49838	2.58068	-3.01843	C	3.63465	1.55866	3.47870
C	2.04971	3.81399	-2.89530	C	4.37194	1.50446	2.34950
C	1.91363	4.45311	-1.69317	C	3.84025	1.06901	1.13314
C	1.19101	3.86360	-0.67272	H	4.32445	1.09462	0.31619
H	1.07815	4.31626	0.15485	H	5.28155	1.77433	2.39735
H	2.31602	5.30315	-1.55891	H	4.03127	1.84960	4.29144
H	2.51029	4.20381	-3.62906	C	3.77616	-1.38184	-0.77920
H	1.65043	2.15171	-3.85232	C	4.30552	-2.11955	-1.81869
C	-1.29811	3.74086	1.13807	C	3.62092	-2.17498	-3.02327
C	-1.88366	4.24239	2.26314	C	2.45333	-1.49187	-3.11403

C	-1.85282	3.54134	3.47023	H	2.01500	-1.54836	-3.95497
H	-2.19876	3.93650	4.26185	H	3.94198	-2.66807	-3.76911
H	-2.32333	5.08406	2.23494	H	5.12718	-2.58551	-1.71743
H	-1.32084	4.23917	0.32957	H	4.23777	-1.32956	0.04947
C	-3.53385	1.86950	5.40040	N	-0.71687	-1.89047	-2.13584
C	-4.38505	1.84493	6.44298	C	-0.66754	-2.58135	-0.95766
C	-3.96081	1.38649	7.67667	N	0.00420	-1.89111	0.07043
C	-2.68089	0.92654	7.76141	C	0.58310	-2.55602	1.16692
H	-2.41909	0.58936	8.61010	N	0.66554	-1.76790	2.26827
H	-4.55396	1.40138	8.41859	C	1.19349	-2.28396	3.43096
H	-5.28024	2.14312	6.33244	N	1.24012	-1.42731	4.49330
H	-3.84671	2.17140	4.55573	C	2.26177	-1.45408	5.43602
N	-1.93473	0.84000	-2.05589	N	1.95396	-0.87922	6.62619
C	-2.57297	0.72698	-0.88392	C	2.93434	-0.80423	7.55902
N	-1.89204	-0.02509	0.10162	C	4.21470	-1.21638	7.34579
C	-2.52937	-0.62437	1.16580	C	4.53304	-1.76202	6.09321
N	-1.76031	-0.68664	2.32430	C	3.54583	-1.86725	5.13950
C	-2.26609	-1.29805	3.44450	H	3.74431	-2.22199	4.28086
N	-1.40220	-1.27064	4.54487	H	5.41351	-2.05809	5.89418
C	-1.39844	-2.24869	5.50639	H	4.88403	-1.14308	8.01595
N	-0.81437	-1.88031	6.68726	H	2.74530	-0.44807	8.41920
C	-0.73901	-2.79048	7.66763	C	1.55206	-3.61743	3.48933
C	-1.17599	-4.04092	7.55948	C	1.51272	-4.37213	2.33918
C	-1.73679	-4.46380	6.35928	C	1.08602	-3.84000	1.12437
C	-1.83750	-3.55226	5.34970	H	1.12559	-4.30751	0.29831
H	-2.21910	-3.82906	4.52491	H	1.78644	-5.28064	2.38594
H	-2.04554	-5.35224	6.22571	H	1.82303	-4.01429	4.30883
H	-1.10857	-4.64365	8.29070	C	-1.28577	-3.79738	-0.82230
H	-0.35412	-2.55683	8.50415	C	-1.90805	-4.40140	-1.87434
C	-3.54706	-1.80174	3.44413	C	-1.83888	-3.76077	-3.09608
C	-4.29362	-1.73027	2.28983	C	-1.28978	-2.53676	-3.17973
C	-3.78501	-1.19427	1.14094	H	-1.31989	-2.12437	-4.03502

H	-4.28349	-1.21022	0.33238	H	-2.17821	-4.18296	-3.87653
H	-5.18300	-2.06401	2.30119	H	-2.37434	-5.22536	-1.79578
H	-3.91740	-2.19464	4.22578	H	-1.27398	-4.21953	0.02867
C	-3.82182	1.34502	-0.64758	Au	0.73741	2.49678	-9.02360
C	-4.36007	2.11566	-1.65993	Au	-1.96551	1.50315	-9.05982
C	-3.72938	2.22866	-2.85028	Au	0.16385	-2.57928	13.59234
C	-2.51437	1.60738	-3.02752	Au	2.54082	-0.95332	13.56710
H	-2.07677	1.72734	-3.86215				

Table S2. Calculated orbital energies of $\mathbf{1}_{AFM}$ and $\mathbf{1}_{FM}$ systems (in eV).

	$\mathbf{1}_{AFM}$		$\mathbf{1}_{FM}$	
	Alpha	Beta	Alpha	Beta
LUMO+9	-1.243	-1.242	-1.187	-1.395
LUMO+8	-1.415	-1.419	-1.253	-1.460
LUMO+7	-1.473	-1.473	-1.433	-1.475
LUMO+6	-1.504	-1.483	-1.481	-1.593
LUMO+5	-1.619	-1.627	-1.509	-1.730
LUMO+4	-1.760	-1.786	-1.645	-1.793
LUMO+3	-1.913	-1.916	-1.820	-1.879
LUMO+2	-1.952	-1.931	-1.963	-2.044
LUMO+1	-2.626	-2.631	-2.632	-2.627
LUMO	-2.657	-2.651	-2.657	-2.652
HOMO	-4.889	-4.886	-4.886	-4.890
HOMO-1	-4.893	-4.896	-4.894	-4.897
HOMO-2	-5.257	-5.263	-5.291	-5.216
HOMO-3	-5.429	-5.430	-5.437	-5.418
HOMO-4	-5.464	-5.468	-5.475	-5.453
HOMO-5	-5.478	-5.481	-5.485	-5.466
HOMO-6	-5.531	-5.550	-5.550	-5.529
HOMO-7	-5.581	-5.559	-5.563	-5.549
HOMO-8	-5.657	-5.642	-5.581	-5.696
HOMO-9	-5.710	-5.723	-5.730	-5.714

Table S3. Calculated $g_{L1}g_{MR}$ and site-overlap values [a)] of **1**$_{\mathbf{AFM}}$ and **1**$_{\mathbf{FM}}$ systems (in a.u.).

	**1**$_{\mathbf{AFM}}$		**1**$_{\mathbf{FM}}$	
	Alpha	Beta	Alpha	Beta
$g_{L1}g_{MR}$	0.145	0.174	0.154	0.163
Site-overlap				
LUMO+9	0.000	0.000	0.000	0.000
LUMO+8	0.000	0.000	0.000	0.000
LUMO+7	0.000	0.000	0.000	0.000
LUMO+6	0.000	0.000	0.000	0.000
LUMO+5	0.000	0.000	0.000	0.000
LUMO+4	0.000	0.000	0.000	0.000
LUMO+3	0.000	0.000	0.000	0.000
LUMO+2	0.000	0.000	0.000	0.000
LUMO+1	0.000	0.000	0.000	0.000
LUMO	0.000	0.000	0.000	0.000
HOMO	0.000	0.000	0.000	0.000
HOMO-1	0.000	0.000	0.000	0.000
HOMO-2	0.000	0.000	0.000	0.000
HOMO-3	0.000	0.000	0.000	0.000
HOMO-4	0.000	0.000	0.000	0.000
HOMO-5	0.000	0.000	0.000	0.000
HOMO-6	0.000	0.006	0.159	0.000
HOMO-7	0.000	0.004	0.213	0.000
HOMO-8	0.034	0.015	0.003	0.092
HOMO-9	0.225	0.368	0.448	0.146

a) The site-overlap was obtained with the overlap matrix in terms of the atomic orbital basis functions printed in the output file using the IOP(3/33=4).

Table S4. Calculated atomic spin densities of $\mathbf{1}_{\mathbf{AFM}}$ and $\mathbf{1}_{\mathbf{FM}}$ systems (in atomic unit). The order of atoms corresponds to Table S1.

	Atom	AFM	FM	Fragments		Atom	AFM	FM	Fragments
1	Ni	-1.59	1.59	Ni ions	71	H	0.00	0.00	
2	Ni	-0.11	0.12		72	H	0.00	0.00	
3	Ni	0.00	0.03		73	H	0.00	0.00	
4	Ni	0.11	0.12		74	N	-0.06	0.06	
5	Ni	1.59	1.60		75	C	0.00	0.00	
6	N	-0.06	0.06	NCS ligand	76	N	0.00	0.00	
7	C	0.01	-0.01		77	C	0.00	0.00	
8	S	0.00	0.00		78	N	0.00	0.00	
9	N	0.06	0.06	NCS ligand	79	C	0.00	0.00	
10	C	-0.01	-0.01		80	N	0.00	0.00	
11	S	0.00	0.00		81	C	0.00	0.00	
12	N	0.06	0.06	other ligands	82	N	0.06	0.06	
13	C	0.00	0.00		83	C	-0.01	-0.01	
14	N	0.00	0.00		84	C	0.00	0.00	
15	C	0.00	0.00		85	C	-0.01	-0.01	
16	N	0.00	0.00		86	C	0.00	0.00	
17	C	0.00	0.00		87	H	0.00	0.00	
18	N	0.00	0.00		88	H	0.00	0.00	
19	C	0.00	0.00		89	H	0.00	0.00	
20	N	-0.06	0.06		90	H	0.00	0.00	
21	C	0.01	-0.01		91	C	0.00	0.00	
22	C	0.00	0.00		92	C	0.00	0.00	
23	C	0.01	-0.01		93	C	0.00	0.00	
24	C	0.00	0.00		94	H	0.00	0.00	
25	H	0.00	0.00		95	H	0.00	0.00	
26	H	0.00	0.00		96	H	0.00	0.00	
27	H	0.00	0.00		97	C	0.00	0.00	
28	H	0.00	0.00		98	C	0.01	-0.01	

29	C	0.00	0.00	99	C	0.00	0.00
30	C	0.00	0.00	100	C	0.01	-0.01
31	C	0.00	0.00	101	H	0.00	0.00
32	H	0.00	0.00	102	H	0.00	0.00
33	H	0.00	0.00	103	H	0.00	0.00
34	H	0.00	0.00	104	H	0.00	0.00
35	C	0.00	0.00	105	N	-0.06	0.06
36	C	0.00	0.00	106	C	0.00	0.00
37	C	0.00	0.00	107	N	0.00	0.00
38	C	-0.01	-0.01	108	C	0.00	0.00
39	H	0.00	0.00	109	N	0.00	0.00
40	H	0.00	0.00	110	C	0.00	0.00
41	H	0.00	0.00	111	N	0.00	0.00
42	H	0.00	0.00	112	C	0.00	0.00
43	N	-0.05	0.05	113	N	0.06	0.06
44	C	0.00	0.00	114	C	-0.01	-0.01
45	N	0.00	0.00	115	C	0.00	0.00
46	C	0.00	0.00	116	C	-0.01	-0.01
47	N	0.00	0.00	117	C	0.00	0.00
48	C	0.00	0.00	118	H	0.00	0.00
49	N	0.00	0.00	119	H	0.00	0.00
50	C	0.00	0.00	120	H	0.00	0.00
51	N	0.05	0.05	121	H	0.00	0.00
52	C	-0.01	-0.01	122	C	0.00	0.00
53	C	0.00	0.00	123	C	0.00	0.00
54	C	-0.01	-0.01	124	C	0.00	0.00
55	C	0.00	0.00	125	H	0.00	0.00
56	H	0.00	0.00	126	H	0.00	0.00
57	H	0.00	0.00	127	H	0.00	0.00
58	H	0.00	0.00	128	C	0.00	0.00
59	H	0.00	0.00	129	C	0.01	-0.01
60	C	0.00	0.00	130	C	0.00	0.00

61	C	0.00	0.00	131	C	0.01	-0.01	
62	C	0.00	0.00	132	H	0.00	0.00	
63	H	0.00	0.00	133	H	0.00	0.00	
64	H	0.00	0.00	134	H	0.00	0.00	
65	H	0.00	0.00	135	H	0.00	0.00	
66	C	0.00	0.00	136	Au	-0.03	0.00	Electrodes
67	C	0.00	0.00	137	Au	0.04	0.00	
68	C	0.00	0.00	138	Au	0.00	0.00	
69	C	0.01	-0.01	139	Au	0.00	0.00	
70	H	0.00	0.00					

	AFM			AFM	
	Alpha	Beta		Alpha	Beta
HOMO	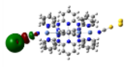	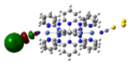	LUMO+9	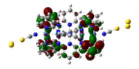	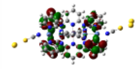
HOMO-1	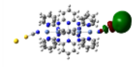	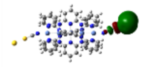	LUMO+8	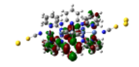	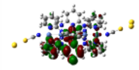
HOMO-2	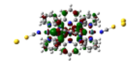	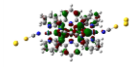	LUMO+7	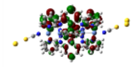	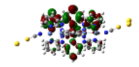
HOMO-3	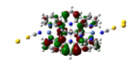	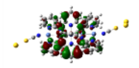	LUMO+6	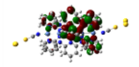	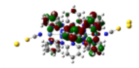
HOMO-4	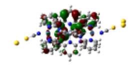	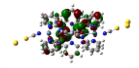	LUMO+5	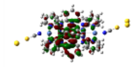	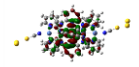
HOMO-5	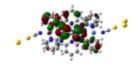	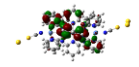	LUMO+4	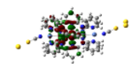	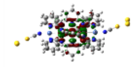
HOMO-6	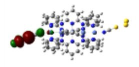	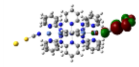	LUMO+3	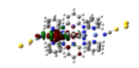	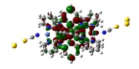
HOMO-7	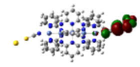	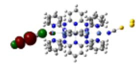	LUMO+2	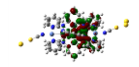	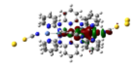
HOMO-8	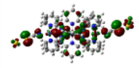	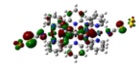	LUMO+1	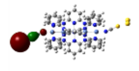	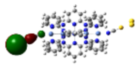
HOMO-9	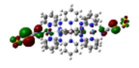	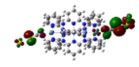	LUMO	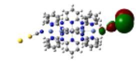	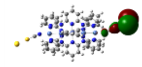

(A)

Figure S1. Illustrations of LUMO+9 to HOMO-9 of (A) $\mathbf{1}_{\mathbf{AFM}}$ and (B) $\mathbf{1}_{\mathbf{FM}}$.

Figure S1 continues to the next page.

	FM			FM	
	Alpha	Beta		Alpha	Beta
HOMO	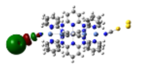	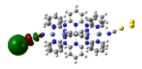	LUMO+9	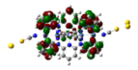	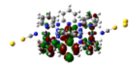
HOMO-1	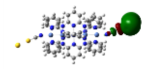	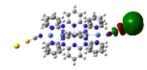	LUMO+8	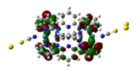	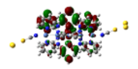
HOMO-2	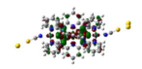	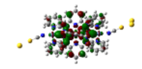	LUMO+7	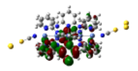	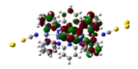
HOMO-3	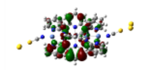	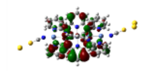	LUMO+6	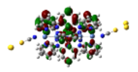	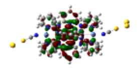
HOMO-4	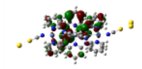	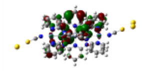	LUMO+5	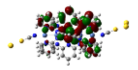	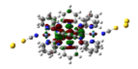
HOMO-5	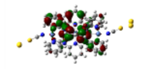	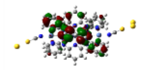	LUMO+4	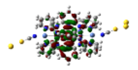	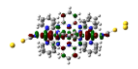
HOMO-6	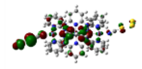	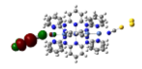	LUMO+3	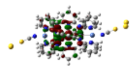	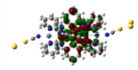
HOMO-7	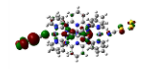	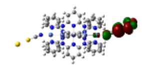	LUMO+2	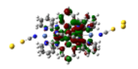	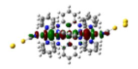
HOMO-8	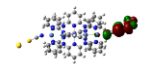	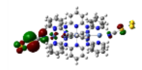	LUMO+1	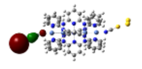	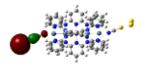
HOMO-9	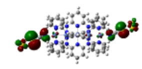	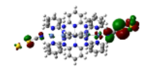	LUMO	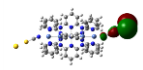	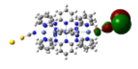

(B)

Figure S1. Illustrations of LUMO+9 to HOMO-9 of (A) **1**$_{\mathbf{AFM}}$ and (B) **1**$_{\mathbf{FM}}$.
